# Emotion Regulation in Close Relationships: The Role of Individual Differences and Situational Context

**DOI:** 10.3389/fpsyg.2021.697901

**Published:** 2021-07-28

**Authors:** Wan-Lan Chen, Wan Ting Liao

**Affiliations:** ^1^Department of Human Development and Psychology, Tzu Chi University, Hualien City, Taiwan; ^2^Taipei Veterans General Hospital, Taipei, Taiwan

**Keywords:** emotion regulation, close relationships, situational context, perception of control, relational goal, emotion intelligence

## Abstract

A substantial amount of research has examined the role of individual differences in the regulation of emotion and the impact of emotion regulation on mental health; however, few studies have covered the role of situational context in the selection of emotion regulation strategies. In this paper, we investigate the extent to which an individual’s choice of emotion regulation strategy is affected by factors such as emotional intelligence, the person with whom one is in conflict, situational sense of control, and the individual’s aim in dealing with the conflict. A total of 300 participants (46.67% female) between the ages of 21 and 35 were recruited from the community (female’s mean age = 28.14, SD = 4.49; male’s mean age = 28.12, SD = 4.32). Participants filled out a set of questionnaires related to their emotion intelligence and emotion regulations they used in two interpersonal incidents with parents and partner. Structural equation modeling was used for data analyses. Results showed that positive correlation between emotional intelligence and cognitive reappraisal, in contrast to previous studies, a positive correlation between emotional intelligence and repression was found. Moreover, the person one is interacting with influences the degree to which one’s sense of control impacts the choice of emotion regulation strategy. For example, in the event of conflict with one’s parents, the degree of situational control has little impact on emotion regulation; however, in conflicts with spouses or partners, women have more situational control and are more likely to use cognitive reappraisal or suppression. Regarding the relationship between the goal of emotion regulation and the strategies used, this study found that they are moderated by gender and the persons involved; for example, when maintaining the relationship is the primary goal of emotion regulation, cognitive reappraisal is more likely the strategy of choice for men involved in a conflict with their partner and for women involved in a conflict with their parents. Overall, the results confirm that emotion regulation is affected by both individual and situational factors, indicating the importance of adopting a dynamic approach when investigating emotion regulation.

## Introduction

The establishment of close interpersonal relationships is an important task in early adulthood (Conger et al., 2000; Dinero et al., 2008), and maintaining stable close relationships at this stage of life has been found to influence psychological health during later life stages (Kiecolt-Glaser and Newton, 2001; Schulenberg et al., 2004; Lehnart et al., 2010). Emotion plays an important role in our relationships (Keltner and Haidt, 1999), and a close relational context in particular shapes our experience of emotion. For example, when a son expresses apprehension over an upcoming exam, his parents can respond adequately by validating his feeling. Such exchanges between the son and his parents may strengthen their relationship. A woman who feels she is perceived negatively by her intimate partner may become increasingly dissatisfied with the relationship. Studies have found that how we deal with conflict in a relationship and how we regulate the emotions resulting from those conflicts affect not only the quality and longevity of the relationship (Zaki and Williams, 2013; Marroquín and Nolen-Hoeksema, 2015) but also our mental health (Duvila et al., 2003; Fingerman et al., 2008).

Individual differences, such as attachment style (Pace et al., 2016) personality traits (Ong et al., 2006; Ng and Diener, 2009; Eldesouky and English, 2019), emotional intelligence (EI) (Bucich and MacCann, 2019), gender (Nolen-Hoeksema and Aldao, 2011; Kwon et al., 2013), and cultural background (Butler et al., 2007; Matsumoto et al., 2008) may influence the use of a particular emotion regulation strategy. For example, individuals with high EI are more likely to use cognitive reappraisal (Bucich and MacCann, 2019), women tend to employ a greater variety of emotion regulation strategies than men do (Nolen-Hoeksema and Aldao, 2011), and individuals in cultures that emphasize harmonious social relationships tend to suppress negative emotions (Butler et al., 2007).

Among the numerous previous studies on the impact of individual and contextual factors on emotion regulation, few have considered situational factors. The many factors found to affect interpersonal interactions include the person with whom one is interacting and one’s sense of control in the situation (Troy et al., 2013) and the goal of emotion regulation (English et al., 2017), all of which may affect an individual’s choice of emotion regulation strategy. In discussions of various emotion regulation techniques, it is helpful to see each one as an integral whole, used by a particular kind of person (personality traits, etc.) in a specific situation (the people involved, the level of control, etc.) and flexibly applied to deal with stress and solve problems (Bonanno et al., 2004; Sheppes et al., 2011; Aldao and Nolen-Hoeksema, 2012). Surprisingly, very few studies have touched on the impact of situational factors on an individual’s choice of emotion regulation strategy (Wenzel et al., 2019). Thus, the purpose of the present study is to investigate from a dynamic perspective the choice of an emotion regulation strategy when conflict occurs in a close relationship, taking into account both individual factors and the overall context, including the people involved, level of control, and goal of emotion regulation.

### Emotion Regulation

In the process model of emotion regulation, emotion regulation refers to the process by which an individual consciously or unconsciously monitors and adjusts his or her emotions (Gross and John, 2003). Emotion regulation can be divided into two types. The first type is when the individual responds to changes in the external environment or in his or her internal mental state by adjusting his or her emotions. This type occurs at the stage of emotion generation, so it is called antecedent-focused emotion regulation and includes situation selection, situation modification, attentional deployment, and cognitive change. The other type is when the individual weakens or enhances his or her emotional response by means such as expressive suppression, exercise, drugs, or relaxation training. This type occurs after the emotional response is generated, so it is called response-focused emotion regulation (Gross, 1998). Among the various means of emotion regulation, the two that have received the most attention have been reappraisal (a form of antecedent-focused emotion regulation) and suppression (a form of response-focused emotion regulation).

Cognitive reappraisal refers to modifying the significance one assigns to an event so as to reduce its emotional impact; for example, one may tell himself or herself, “This won’t be easy, but I’ll give it a try, and if I encounter any difficulties, I’ll find a way to solve them.” By contrast, suppression refers to holding one’s emotional reaction in check while experiencing that emotion, for example, feeling frustrated inside but inhibiting its corresponding outward expression (Gross and Levenson, 1993; Gross, 1998). Studies have found that people who tend to adopt avoidance and suppression in response to negative emotions are more likely to experience psychological problems (Pace and Muzi, 2017; Velotti and Rogier, 2021). Conversely, individuals who are able to reappraise emotional events or take positive actions to deal with problematic situations are better at adapting to the vicissitudes of life (Mauss et al., 2007; Hofmann et al., 2012; Webb et al., 2012; Carl et al., 2013; Pace et al., 2018).

While the process model provides a simple and clear explanation of emotion regulation, it fails to take into account various contextual factors found to influence which emotion regulation strategies an individual tends to use (Young and Suri, 2019). For example, in a conflict with one’s partner or in a situation in which one has a sense of situational control or is otherwise motivated to exercise emotion regulation, he or she is more likely to use cognitive reappraisal. However, in another situation, the individual may intentionally choose to refrain from controlling negative emotions, thereby highlighting the conflict, to make it necessary to face the problem. The above examples show that an individual’s valuation of the situation affects his or her mode of emotion regulation, and the difference between the results of emotion regulation and the expected outcome may affect one’s subsequent use of emotion regulation, forming an interactive process between the individual and the situation that continually evolves over time (Gross, 2015). To emphasize this dynamic, research needs to systematically examine individual differences and analyze how different situations affect a person’s choice of emotion regulation strategy.

### Situational Context and Emotion Regulation

In Sheppes et al. (2015) study, they propose that whether emotion regulation would occur depends on individual’s evaluation of current goals or expectation. Recent research on emotion regulation has begun to focus more on the contextual and situational factors at play in emotion regulation (McRae et al., 2011; Troy et al., 2013; Sheppes et al., 2015; Haines et al., 2016; English et al., 2017; Kobyliǹska and Kusev, 2019), including the person one is interacting with and one’s sense of control over the situation. A person’s approach to emotion regulation tends to differ depending on whether they are interacting with a partner or a colleague (Gross et al., 2006), and the less close the relationship, the more likely one is to adopt expressive suppression (English et al., 2017). In Eastern Asia, such as Taiwan, Confucianism had significant influence upon people’s daily lives. In Confucian teaching, interpersonal relationships-close relationships in particular-are characterized by kinship value and respect for seniority (Hill, 2007). We are interested to examine whether the use of emotion regulation varies across different close relationship (such as parents or partner). In addition, one’s level of situational control is significantly correlated with the emotion regulation strategy used (Troy et al., 2013; Haines et al., 2016). When the individual has little control over the situation, cognitive reappraisal tends to be regarded as a more appropriate mode of emotion regulation, in contrast to situations in which the individual has greater control (Cheng, 2001; Troy et al., 2013).

Another situational factor is the goal of emotion regulation; the emotion regulation strategy adopted tends to differ when the primary goal is to make a good impression on the other party, as opposed to a situation in which the chief goal is to foster mutual understanding (English et al., 2017). For example, a person experiencing conflict with his or her parents due to differing views is likely to be socially motivated to suppress his or her anger so as to avoid straining the relationship (Tamir, 2016). However, if the person’s primary motivation is to ward off excessive control by his or her parents, he or she is more likely to express anger and other negative emotions (Tamir and Ford, 2012). One of the central Confucian ideologies is the maintenance of interpersonal harmony (Matsumoto et al., 2008), which is also deeply embedded in the value of Taiwanese society. Therefore, we are interested to investigate the extent to which individuals regulate their emotions during the conflict in order to maintain harmony in their close relationship.

### Individual Differences in Emotion Regulation

How an individual responds to or expresses emotions is affected by socialization (McRae et al., 2011), in which the individual learns gender-compliant modes of emotion regulation (Ryan et al., 2005). Generally, men are expected to not be overly emotional, especially in regard to negative emotions such as sadness and dejection (Sloan, 2012). Furthermore, emotion regulation studies have found that men use repression more often than women in their emotional reactions in daily life (Gross and John, 2003; Campbell-Sills et al., 2006), while women are more likely to use repression as they age (Nolen-Hoeksema and Aldao, 2011). Moreover, women verbally express their feelings more often than men do and are more likely to change their living situation to ease their anger or sadness (Rivers et al., 2007).

Regarding cognitive reappraisal strategies, the findings are more divergent. While some studies have found no significant difference between men and women in terms of cognitive reappraisal (Gross and John, 2003; Gross et al., 2006; McRae et al., 2008), others have found that women use cognitive reappraisal more often than men (McRae et al., 2011). Is it possible that this difference is due to the influence of situational factors on the selection of an emotion regulation strategy? This is a question requiring further research.

In addition to contextual factors, research shows that emotional traits can also partially explain a person’s choice of emotion regulation strategy (Mikolajczak et al., 2008), which is seen by some scholars as a form of EI (Petrides and Furnham, 2003). In an effort to integrate two traditions of emotion research together, namely, emotion regulation and emotion intelligence, to provide a complete theoretical interpretation of emotion, Peña-Sarrionandia et al. (2015) use emotion regulation as a framework to conceptualize individual differences in the process and consequence of emotion regulation. Their review suggests that the use of emotion strategies significantly vary by the level of EI. Research has found that people with higher EI have higher life satisfaction (Di Fabio and Saklofske, 2014), better health (Schutte et al., 2007; Malouff et al., 2014), less emotional distress (Mikolajczak et al., 2014), and better performance at school or work (Van Rooy and Viswesvaran, 2004; O’Boyle et al., 2011).

Goleman (1995) suggested that EI is a crucial component in maintaining relationships. A number of studies have provided empirical evidence to support that individuals with higher EI tended to be more empathic (Mayer et al., 2008) and socially connected (Lopes et al., 2005), and to have better interpersonal relationships (Schutte et al., 2001). Based on this view, it is expected that that EI is positively related to maintenance of harmonious relationships when dealing with conflict with parents or intimate a partner.

According to Peña-Sarrionandia et al. (2015), rather than continuously regulating their emotions, high-EI individuals are better than low-EI individuals at flexibly selecting a suitable emotion regulation strategy when the situation requires it. To be sure, the selection and use of an emotion regulation strategy needs to be adjusted according to the situation, and EI appears to play a role in evaluating the situation and strategy employed.

### The Present Study

The purpose of the present study is to elucidate how emotional competence and situational factors affect the selection of an emotion regulation strategy when dealing with conflict. Taking EI as an indicator of emotional competence, we hypothesize that individuals with higher EI would be better able to reevaluate a situation by looking at it from a different perspective and better at using cognitive reappraisal to regulate emotions during interpersonal conflict. We presume that there is a negative correlation between EI and suppression of emotion in response to interpersonal conflict. For situational factors, we expect that both the sense of control over the conflict situation and the goal of dealing with the conflict influence one’s selection of an emotion regulation strategy. When an individual has a high sense of control, we predict that he or she is more likely to take action to change the current situation and therefore less likely to use cognitive reappraisal or to suppress emotion. By contrast, if the individual sees maintaining the relationship as the goal of dealing with the conflict, he or she is more likely to use cognitive reappraisal and suppression. In this study, we focus on the extent to which the impact of situational factors on emotion regulation is influenced by gender and the person one is interacting with (parents vs. partners).

## Materials and Methods

### Participants

A total of 300 participants between the ages of 21 and 35 were recruited from the community. Of these, 140 were women (mean age = 28.14, *SD* = 4.49), and 160 were men (mean age = 28.12, *SD* = 4.32). The majority of the sample had a college degree (60%), high school education (21%), and a graduate degree (19%). A large percentage of participants were single (71.0%), and another 27% were married. Approximately two thirds of the participants worked in a full-time job, including services and sales (18.7%), technical (14.7%), professional (12.2%), clerical (10%), and education (9.5%).

Potential participants were excluded if they (a) were below age 20 or above age 35, (b) had no in-person contact with their parents for the past 3 months, or (c) had not been in an intimate relationship during the past 3 months.

### Procedure

After signing an informed consent form, participants were asked to complete a set of questionnaires including background information and measures of EI. Then, they were instructed to recall a recent conflict in which they experienced anger with (1) one of their parents and (2) an intimate partner. Next, participants were asked to evaluate their emotion regulation, perception of control over the situation, and goal of emotion regulation during that experience. Each participant was given a voucher worth $10 upon completion. This research was approved by the Research Ethics Committee of Hualien Tzu Chi Hospital, Buddhist Tzu Chi Medical Foundation (no. IRB106-72-B).

### Measures

#### Emotion Intelligence

The Wong and Low Emotion Intelligence Scale (WLEIS; Wong and Law, 2002), a self-report scale, consists of four dimensions of trait EI. Each dimension has 4 items, for a total of 16 items. The four dimensions include (1) self-emotional appraisal, e.g., “I have a good sense of why I have certain feelings most of the time”; (2) others’ emotional appraisal, e.g., “I am sensitive to the feeling and emotions of others”; (3) use of emotion, e.g., “I am able to control my temper and handle difficulties rationally”; and (4) regulation of emotion, e.g., “I have good control of my own emotions.” The WLEIS is answered on a five-point Likert scale ranging from 1 (strongly disagree) to 5 (strongly agree). Prior research indicates that the WLEIS has good reliability and convergent and predictive validity (e.g., Fukuda et al., 2012; Li et al., 2012; Libbrecht et al., 2014; LaPalme et al., 2016). Cronbach’s alphas for the scales of WLEIS in the present study range from 0.81 to 0.90.

#### Context-Specific Emotion Regulation

Participants were asked to recall a time during the past 3 months when they had felt anger resulting from a conflict with (1) their parents or (2) their intimate partner, respectively. After briefly describing each incident, the Emotion Regulation Questionnaire (ERQ; Gross and John, 2003) was used to assess the emotion regulation associated with the incident. The instructions given to the participants were: “We would like to ask you some questions about how you regulate your emotion during the conflict with your parents/partner, the questions below involve two distinct aspects of your emotion life. Please answer using the following scale.”

The ERQ is a 10-item measure designed to assess cognitive reappraisal with six items (e.g., “When I want to feel more positive emotion, I change the way I’m thinking about the situation”) and emotion suppression with 4 items (“I control my emotions by not expressing them”). Participants were asked to rate their emotion regulation in the two incidents with parents and partner, respectively. Each item was rated on a scale from 1 (*strongly disagree*) to 7 (*strongly agree*), and the total score for each strategy was divided by the number of items, with higher scores indicating a greater tendency to use the strategy to regulate emotion. Cronbach’s alphas for cognitive reappraisal in the present study were 0.89 and 0.91 for parents and intimate partners, respectively; for suppression, they were 0.75 and 0.79 for parents and intimate partners, respectively.

#### Perception of Control Over the Conflict

Perception of controllability was assessed by two items. Participants rated their sense of control over the (1) cause and (2) outcome of the conflict in each interpersonal context on a scale from 1 (not at all) to 7 (a great deal). The scale has good internal reliability, and Cronbach’s alphas were 0.86 and 0.85 for parents and intimate partners, respectively.

#### Relational Goal of Emotion Regulation

Participants indicated the extent to which they regulated their emotions during the conflict in order to maintain a positive relationship (1 = *not at all* to 7 = *a great deal*).

Relational goals of emotion regulation for parents and intimate partners were assessed individually.

### Data Analytic Plan

A series of independent and dependent samples *t* tests were conducted to test gender as well as within-person differences on emotion regulations in different interpersonal contexts. We adopted a two-step approach (Anderson and Gerbing, 1988; Anderson and Gerbing, 1992) of structural equation modeling to test the hypothetical models. Confirmatory factor analyses were conducted to examine whether the measurement models fit the data well. After an acceptable measurement model was developed, the structural model was tested. The maximum likelihood estimation in AMOS 20.0 was used to estimate the parameters. Three goodness-of-fit indices recommended by Hu and Bentler (1999) were adopted to evaluate the mode fit: a confirmatory fit index (CFI) close to 0.96, a standardized root mean square residual (SRMR) less than 0.08, and a root mean square error of approximation (RMSEA) less than 0.06. The chi-squared (χ^2^) test is another index to assess model fit, and a non-significant result indicates that the model fits the data adequately; however, this test is easily affected by the sample size. Kline (2005) suggested an acceptable ratio of chi square to degrees of freedom (χ^2^/df). The analyses of multiple-group comparison were conducted to examine the invariance of path coefficients between gender differences. In one model, the path coefficients were constrained to be equal for female and male, and in the other model the path coefficients were allowed to vary. A chi-square test was examined to determine whether these two models were equivalent. A bootstrap method was adopted to verify the significance of indirect effect the structural model (MacKinnon et al., 2002).

## Results

Table 1 presents the means and standard deviations of the study variables for males and females. The results of the independent-sample *t*-test indicated that males were more likely than females to regulate their anger by using suppression in both interpersonal situations. We also compared reappraisal and suppression in the same interpersonal context by conducting dependent-sample *t*-tests to examine possible within-participant differences. The results showed that individuals tended to regulate anger by using more reappraisal than suppression in their close relationships, whether with parents (female, *t*(139) = 7.08, *p* < 0.001); male, *t*(159) = 6.73, *p* < 0.001) or with an intimate partner (female, *t*(139) = 8.28, *p* < 0.001); male, *t*(159) = 6.39, *p* < 0.001).

**TABLE 1:** Means and standard deviations of the research variables for male and female groups.

**Variable**	**Female (*n* = 140)**		**Male (*n* = 160)**		
	***M***	***SD***	***M***	***SD***	***t*(298)**
Emotion intelligence	57.32	10.14	59.69	10.87	–1.94
**Parents**					
Reappraisal	4.95	0.94	5.03	1.00	−72
Suppression	4.32	1.10	4.57	1.08	−1.98*
Controllability	9.17	2.94	9.52	2.69	–1.07
Relational goal	5.76	1.15	5.60	1.18	1.17
**Intimate partner**					
Reappraisal	4.96	1.08	5.18	0.99	–1.80
Suppression	4.21	1.20	4.70	1.18	−3.54***
Controllability	9.25	2.86	9.78	2.81	–1.62
Relational goal	5.90	1.20	5.81	1.16	0.64

*p < 0.05, ***p < 0.001.

### Measurement Model

The latent variables with unidimensional measures (such as appraisal) were estimated using the item parceling technique (Russell et al., 1998). The items were ranked from high to low according to the corrected item-total correlation and then evenly distributed to three parcels for greater consistency of variance. The EI measurement indices were the scores of its four subscales, namely, self-emotion appraisal, others’ emotion appraisal, use of emotion, and regulation of emotion. The measurement models were tested separately for the two types of close relationships. Each measurement model comprised three latent variables (see Table 2) and two measurement variables, and there were 12 observation indices.

**TABLE 2:** Factor loadings for the Measurement Model.

**Latent variable and indicator**	***ß***	***SE***	**Z**	**95% CI**
**Emotion intelligence**				
Self-emotional appraisal	0.80***			(0.74, 0.85)
Others’ emotional appraisal	0.79***	0.076	14.67	(0.73, 0.84)
Use of emotion	0.74***	0.078	13.58	(0.67, 0.80)
Regulation of emotion	0.88***	0.077	16.55	(0.83, 0.92)
**Parents appraisal**				
Parcel 1	0.87***			(0.82, 0.91)
Parcel 2	0.89***	0.048	19.99	(0.84, 0.93)
Parcel 3	0.80***	0.056	17.06	(0.72, 0.87)
**Partner suppression**				
Item 2	0.72***			(0.62, 0.82)
Item 4	0.73***	0.083	10.58	(0.73, 0.83)
Item 6	0.82***	0.094	12.48	(0.75, 0.88)
Item 9	0.82***	0.085	12.45	(0.74, 0.88)
**Partner appraisal**				
Parcel 1	0.88***			(0.84, 0.92)
Parcel 2	0.85***	0.048	19.34	(0.79, 0.90)
Parcel 3	0.90***	0.051	21.29	(0.84, 0.94)
**Partner suppression**				
Item 2	0.78***			(0.69, 0.85)
Item 4	0.76***	0.074	11.73	(0.68, 0.83)
Item 6	0.80***	0.075	13.48	(0.71, 0.87)
Item 9	0.82***	0.074	17.74	(0.74, 0.89)

95% CI is presented for standardized factor loadings. ***p < 0.001.

The results indicated that the two models fit the data well parent model, (χ^2^ (46; *N* = 300) = 97.28; *p* < 0.00; χ^2^/df = 2.12; CFI = 0.97; RMSEA = 0.06; SRMR = 0.04; partner model, (χ^2^ (46; *N* = 300) = 126.04; *p* < 0.00; χ^2^/df = 2.74; CFI = 0.96; RMSEA = 0.07; SRMR = 0.04. The loadings of measured indicators on the latent variables were all statistically significant, and the coefficients of composite reliability of latent variables were all greater than 0.6, as suggested by Bagozzi and Yi (1988). All correlations among the variables within these two models were significant (see Table 3).

**TABLE 3:** Correlations among variables in the measurement model.

**Latent variable**	**1**	**2**	**3**	**4**	**5**
1. Emotion intelligence		0.34**	0.40**	0.68**	0.43**
2. Controllability	0.36**		0.47**	0.37**	0.14*
3. Relational goal	0.30**	0.41**		0.53*	0.19**
4. Reappraisal	0.51**	0.46**	0.42**		0.62**
5. Suppression	0.30**	0.28**	0.18**	0.63**	

Correlations above the diagonal are for parents model; correlations below the diagonal are for intimate partner model. *p < 0.05, **p < 0.01.

### Structural Model

**FIGURE 1:**
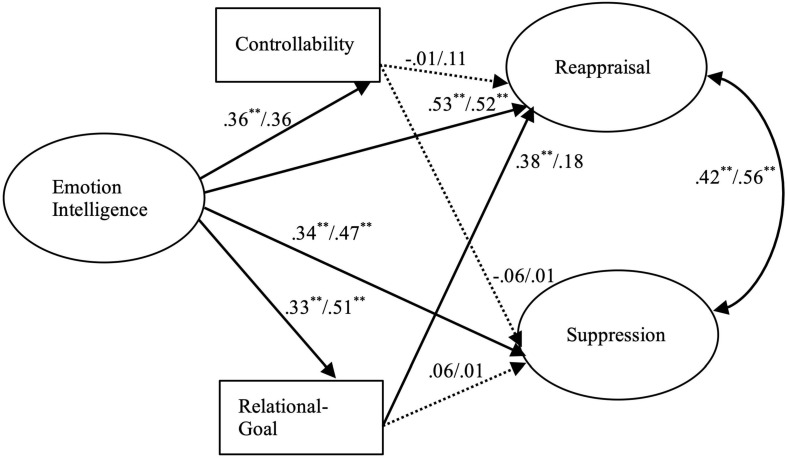
The structural mode of emotion regulation with parents. Dashed lines indicate non-significant paths. The values are the path coefficients for females (left side) and males (right side). *N* = 300. ^∗^*p* < 0.05. ^∗∗^*p* < 0.01.

**FIGURE 2:**
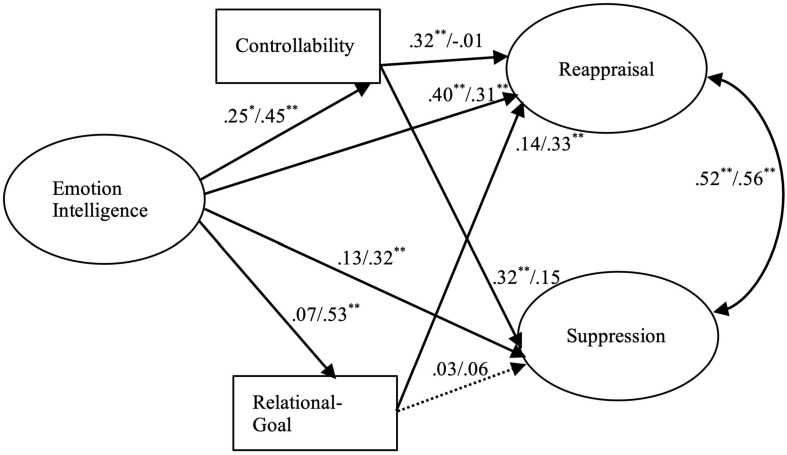
The structural mode of emotion regulation with partners. Dashed lines indicate non-significant paths. The values are the path coefficients for females (left side) and males (right side). *N* = 300. ^∗^*p* < 0.05. ^∗∗^*p* < 0.01.

The results of our hypothetical models (see Figures 1, 2) showed good fits of the models to the data [parent model, χ^2^ (48; *N* = 300) = 143.85; *p* < 0.00; χ^2^/df = 2.99; CFI = 0.97; RMSEA = 0.05; SRMR = 0.03; partner model, χ^2^ (48; *N* = 300) = 140.93; *p* < 0.00; χ^2^/df = 2.93; CFI = 0.96; RMSEA = 0.06; SRMR = 0.04]. A moderating effect of gender (female vs. male) was also evaluated. Comparisons of the unconstrained and constrained models using chi-square differences showed significant results parent model, Δχ^2^(df = 6) = 13.57, *p* < 0.05; partner model, Δχ^2^(df = 6) = 14.74, *p* < 0.05. The results indicated that the coefficients across groups were not equal for males and females. The standardized coefficients are presented in Figure 1 (parent model) and Figure 2 (partner model). Additionally, indirect effects of EI on emotion regulation strategies through a sense of controllability and goals for conflict management were tested. The bootstrap resampling method (MacKinnon et al., 2002) was used by taking a sample of the original data to obtain 1,000 samples to calculate the 95% confidence intervals (CIs) of indirect effects.

The bootstrap results indicated that EI had a significant indirect effect on reappraisal through the sense of controllability and goal for conflict management in the parent model (female β = 0.12, SE = 0.05, 95% CI = 0.05, 0.22; male β = 0.13, SE = 0.05, 95% CI = 0.04, 0.22) and partner model (female β = 0.09, SE = 0.05, 95% CI = 0.01, 0.18; male β = 0.24, SE = 0.06, 95% CI = 0.04, 0.22), whereas EI had a significant indirect effect on suppression through the sense of controllability and goal for conflict management only for females in relations with partners (β = 0.08, SE = 0.04, 95% CI = 0.03, 0.16). EI did not have significant effects on suppression in the parent model (female β = 0.01, SE = 0.05, 95% CI = 0.01, 0.07; male β = 0.13, SE = 0.05, 95% CI = -0.08, 0.10) or partner model for males (β = 0.01, SE = 0.05, 95% CI = -0.08, 0.00).

## Discussion and Conclusion

This study explores how an individual’s choice of emotion regulation strategy in a conflict situation is influenced by factors such as EI, the person one is interacting with, perceived level of control, and the individual’s primary goal in dealing with the conflict. The results showed that in dealing with conflict with their partner or parents, the participants used cognitive reappraisal much more frequently than suppression, and the men used suppression more frequently than the women. Both of these findings are consistent with the results of most previous studies (Brody and Hall, 1993; Gross and John, 2003; Matsumoto et al., 2016; Goubet and Chrysikou, 2019), including previous research that found that in various types of situations, men are much more likely to use expressive suppression (Goubet and Chrysikou, 2019). However, our results differ from those of Nolen-Hoeksema and Aldao (2011), who found no significant correlation between gender and use of repression in their community sample. This discrepancy may have arisen because Nolen-Hoeksema and Aldao’s definition of suppression consisted of emotional, thought, and expressive suppression, whereas in the present study and other studies with similar results (e.g., Gross and John, 2003; Matsumoto et al., 2016), the measurement of suppression included only expressive suppression, leaving the question of gender differences in terms of emotional and thought suppression as a matter requiring further study.

When we compare our results with previous studies that have also used the ERQ, the participants’ scores for expressive suppression were significantly higher than those of clinical and non-clinical samples in Western countries (Joormann and Gotlib, 2010; Ludwig et al., 2020) but similar to those of other non-Western samples (Matsumoto et al., 2016; Zhou et al., 2016). Studies have shown that, in comparison with West, East Asian societies give more importance to rules for emotion display (Matsumoto et al., 1998; Safdar et al., 2009). The suppression of negative emotion in certain situations is often aimed at protecting the feelings of others (Butler et al., 2007; Ford and Mauss, 2015), and those who are adept at suppressing the overt expression of negative emotions are typically esteemed for their self-control and good social skills (Zhou et al., 2016). In addition, suppression of negative emotion is a reliable predictor of better interpersonal skills (Wei et al., 2013); conversely, the suppression of positive emotions is a good predictor of poor psychosocial skills (Zhou et al., 2016).

As expected, we found a positive correlation between EI and cognitive reappraisal, indicating that those with higher EI are better at reassessing problems and looking at a situation from a different point of view; examples include reducing negative emotions by recasting a negative situation in a positive light or by reevaluating a stressful situation from a different perspective. This finding supports those of several previous studies (Mikolajczak and Luminet, 2008; Schutte et al., 2009; Moradi et al., 2011). In contrast to previous studies, we found a positive correlation between EI and the use of expressive suppression, except among women dealing with conflict in an intimate relationship. The negative correlation between EI and the use of expressive suppression found in past studies (Mikolajczak et al., 2007; Austin et al., 2008) might have been observed because those with higher EI give more importance to consistency between emotion and its expression (Peña-Sarrionandia et al., 2015), making them less likely to use expressive suppression. However, studies have also found that people with high EI are more flexible in selecting an emotion regulation strategy to suit a particular situational context (English et al., 2017; Kobyliǹska and Kusev, 2019). Notably, members of societies that value interdependence and interpersonal harmony, such as Taiwan, are more likely to suppress negative emotions that arise in close relationships to maintain harmony and avoid straining the relationship (Butler et al., 2007), and this also applies to those with a relatively high degree of EI.

We also found a positive correlation between EI and sense of control in situations of conflict, regardless of whether the conflict is in relation to one’s parents or partner, but the influence of this sense of control on emotion regulation varies depending on the type of interpersonal relationship one is dealing with. In conflicts with one’s parents, sense of control was not significantly correlated with the use of either cognitive reappraisal or expressive suppression. Confucian societies such as Taiwan give overarching importance to family ties and the hierarchical relationship between parents and children, such that when differing points of view give rise to conflict between parents and children, directly refusing to accept parents’ perspective or overtly expressing strong negative emotions are generally considered to be egregiously unfilial behavior (Hwang, 2000). Thus, we conjectured that in any conflict with parents, the participants, regardless of their sense of control, would report exercising a high degree of emotion regulation, including both cognitive reappraisal and expressive suppression.

For the female participants in conflicts with their partners, we found a positive correlation between sense of control and the use of multiple emotion regulation strategies, including cognitive reappraisal and suppression of anger. This result is in contrast to the findings of previous studies reporting that those with a relatively high sense of control over the situation are more likely to engage in problem-solving or other proactive behaviors and less likely to use cognitive reappraisal or expressive suppression (Cheng, 2001; Haines et al., 2016). The positive correlation we found for women between their sense control and use of both cognitive reappraisal and expressive suppression may indicate that an increased sense of control makes them more likely to adopt a proactive strategy, for example, reducing the intensity of an emotion by reevaluating the situation from a different point of view prior to the emergence of the emotion or as soon as it arises. This finding is similar to the findings of Ouwehand et al. (2006) in their study on the influence of situation-specific factors on emotion regulation. The general perception in Chinese societies is that when one is in a relatively favorable position, he or she is expected to make face-saving allowances for others and to avoid overtly expressing negative emotions (Soto et al., 2011).

As expected, a positive association between EI and relational goal was found, except among women dealing with conflict in an intimate relationship. In light of the previous studies reporting that the goal of emotion regulation influences the choice of strategy (Gross and Jazaieri, 2014), we paid particular attention to whether or not the choice of emotion regulation strategy is influenced by the person one is interacting with when the person’s goal is to maintain the relationship. Indeed, we found that cognitive reappraisal is more likely to be used when maintaining the relationship is the goal of emotion regulation, but only in two scenarios: men in conflict with a partner and women in conflict with parents. In this regard, research has found that in both Eastern and Western societies, adult daughters spend more time with their parents than adult sons do (Birditt et al., 2009; Tao, 2014). To maintain a better relationship with their parents, daughters may engage in cognitive reappraisal prior to the emergence of an emotional response so as to reduce negative emotions. We also found that the male participants’ efforts to regulate emotions were mainly aimed at mitigating the effects of conflict in their relationship with their partner, for which purpose they attempted to regulate their emotions relatively early, including by using cognitive reappraisal, to stem negative emotions.

For men in conflict with parents and women in conflict with a partner, the associations between reappraisal and maintenance of harmony in relationship are not significant but in a positive direction. The inconsistent results suggest that it is premature to conclude the effects of specific individual or contextual factors on the choice of emotion regulation strategies. More studies are needed.

This pioneering study takes individual differences and situational factors (sense of control and the goal of emotion regulation) into consideration and comprehensively discusses the choice of emotion regulation strategies, but the results are subject to a number of methodological limitations. First, although the self-reporting scales used for data collection have sound psychometric properties and are widely used by researchers, the participants’ responses could have been skewed by various situational and individual factors, such as mood, level of interest, strength of memory, and answers in accordance with social expectations rather than in accordance with their actual experience; such factors could have affected the accuracy of the results. Thus, it would be desirable for future research to include the behavioral dimension of emotion regulation (Tottenham et al., 2011) by using a measurement tool that combines subjective (self-reporting) and objective (behavioral evaluation) evaluations so as to provide a more objective assessment of emotion regulation.

Second, the study only examined the effect of maintenance of relationship (as the goal of emotion regulation) on the association between EI and emotion regulation strategies. Future study could extend our conceptual model by examining other relevant goals of emotion regulation, such as prohedonic goals (Wilms et al., 2020) or performance goals (Tamir, 2016). Examining more comprehensive goals of emotion regulation is important because it would increase the reliability of outcomes.

Third, this study of young adults sought to determine to what extent their choice of emotion regulation strategy for dealing with conflict in a close relationship is affected by their sense of control of the situation and their goal in dealing with the conflict. Thus, the participants were asked to consider a recent incident in which they felt angry as a result of a conflict with either their parents or partner, and having the participants focus on a particular incident rather than answering in terms of their everyday experience constitutes one of the main contributions of this study. Nonetheless, the participants could have differed significantly in terms of the type of incident, its intensity, and the accuracy of their memories, all of which could have influenced their responses. Thus, we need to be especially cautious about making inferences based on these results. In view of the above research limitations, future studies need to apply more rigorous controls to ensure that the incidents the participants are referring to are similar in nature. It would also be desirable to measure the subjective stress levels experienced by participants in relation to the incident they are referring to. To improve the ecological validity of the measurement (Csikszentmihalyi and Larson, 1987), future research should adopt the experience-sampling method (Mehl and Conner, 2012), in which the participant is asked to record his or her emotional responses to various emotionally salient events that occur throughout the course of a typical day, including the nature of the event and the emotion regulation strategy employed.

An additional limitation of this study is that the participants were all young adults within a fairly narrow age range. Research on emotion regulation has found that age is an important background factor affecting emotion regulation (Urry and Gross, 2010), that young adults have greater emotional variability (Birditt and Fingerman, 2003), and that for older people, negative emotions are less intense and less frequent (Charles et al., 2009). In terms of strategies for regulating negative emotions, older people are more accommodating and accepting, while younger people tend to seek support and spend more time pondering their current situation (Blanchard-Fields and Coats, 2008), so we need to be cautious about extrapolating these results to other age groups. Thus, future studies on this topic should include a broad range of age groups and compare their use of emotion regulation strategies.

In this study, both individual differences and situational factors had a bearing on the participants’ choice of emotion regulation strategy, a finding that has important implications for prevention and intervention strategies in the mental health field. In terms of intervention strategies, mental health professionals who seek to help a client select suitable emotion regulation strategies for dealing with interpersonal conflict need to give due consideration to situational factors such as the level of control, the people involved, and the client’s goal in dealing with the conflict (Mauss and Tamir, 2013). For example, the therapist might want to advise the client to use cognitive reappraisal strategies such as reinterpreting the situation and adopting more positive ways of thinking; the therapist might also advise the client to suppress the expression of negative emotions to maintain interpersonal harmony. For clients who have difficulty expressing emotions appropriately, the therapist should provide guidance on how to display emotion in a way that suits the situation. Clients accustomed to suppressing emotions may require help in recognizing the emotion regulation strategies they tend to use, in exploring the possible reasons for suppressing their emotions, and in considering the positive and negative effects these strategies have on their interpersonal relationships and on their physical and mental health.

In summary, the findings of this study indicate that the choice of an emotion regulation strategy results from the interaction between the individual and the situation. Individual differences such as EI affect how individuals engage in emotion regulation. To better understand the influence of emotion regulation on individual adaptation, future research should examine a wider range of emotion regulation strategies.

## Data Availability Statement

The raw data supporting the conclusions of this article will be made available by the authors, without undue reservation.

## Ethics Statement

The studies involving human participants were reviewed and approved by Research Ethics Committee, Hualien Tzu Chi Hospital, Buddhist Tzu Chi Medical Foundation. The patients/participants provided their written informed consent to participate in this study.

## Author Contributions

W-LC conceptualized the study, conduct the statistical analysis, and wrote the manuscript. WL assisted with the preparation of the manuscript. Both authors contributed to the article and approved the submitted version.

## Conflict of Interest

The authors declare that the research was conducted in the absence of any commercial or financial relationships that could be construed as a potential conflict of interest.

## Publisher’s Note

All claims expressed in this article are solely those of the authors and do not necessarily represent those of their affiliated organizations, or those of the publisher, the editors and the reviewers. Any product that may be evaluated in this article, or claim that may be made by its manufacturer, is not guaranteed or endorsed by the publisher.
